# PAX2GRAPHML: a python library for large-scale regulation network analysis using BioPAX

**DOI:** 10.1093/bioinformatics/btab441

**Published:** 2021-06-15

**Authors:** François Moreews, Hugo Simon, Anne Siegel, Florence Gondret, Emmanuelle Becker

**Affiliations:** Univ Rennes, Inria, CNRS, IRISA, Rennes, France; Pegase, Inrae, Institut Agro, 35590 Saint-Gilles, France; Univ Rennes, Inria, CNRS, IRISA, Rennes, France; Univ Rennes, Inria, CNRS, IRISA, Rennes, France; Pegase, Inrae, Institut Agro, 35590 Saint-Gilles, France; Univ Rennes, Inria, CNRS, IRISA, Rennes, France

## Abstract

**Summary:**

PAX2GRAPHML is an open-source Python library that allows to easily manipulate BioPAX source files as regulated reaction graphs described in.graphml format. The concept of regulated reactions, which allows connecting regulatory, signaling and metabolic levels, has been used. Biochemical reactions and regulatory interactions are homogeneously described by regulated reactions involving substrates, products, activators and inhibitors as elements. PAX2GRAPHML is highly flexible and allows generating graphs of regulated reactions from a single BioPAX source or by combining and filtering BioPAX sources. Supported by the graph exchange format .graphml, the large-scale graphs produced from one or more data sources can be further analyzed with PAX2GRAPHML or standard Python and R graph libraries.

**Availability and implementation:**

https://pax2graphml.genouest.org.

## 1 Introduction

BioPAX is a standard format encoding biological processes like gene regulation, metabolic pathways or signaling events, that facilitates the inter-operability between data sources and network analysis tools. However, this rich knowledge-oriented data format that finely captures the complexity of biological networks cannot be easily handled without appropriated tools. Software have been recently proposed to design, visualize (Babur *et al.*, 2010; Shannon *et al.*, 2003), parse (Turei *et al.*, 2016), validate (Rodchenkov *et al.*, 2013), query (Babur *et al.*, 2014) and analyze BioPAX files. However, an important missing feature to analyze BioPAX data sources is the ability to interpret BioPAX files into graph structures including the role of physical entities as substrate, product or regulator in the reactions.

**Fig. 1.**
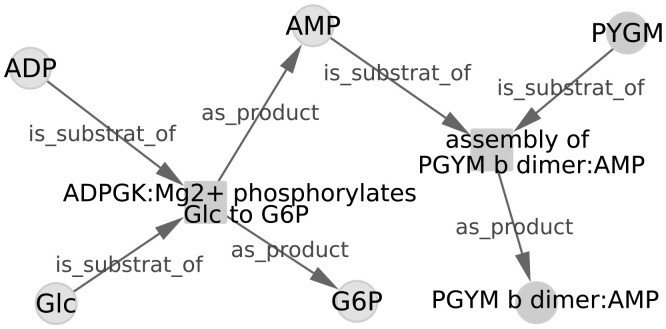
Example of reaction graph manipulated by PAX2GRAPHML showing reactions and entities as nodes

An accurate format for representing the variety and complexity of the biological reactions is the concept of regulated reactions connecting regulatory, signaling and metabolic levels (Blavy *et al.*, 2014). In this conceptual framework, both biochemical reactions and regulatory interactions are described homogeneously as regulated reactions involving substrates, products, activators, inhibitors and modulators as key elements. In the reaction graph generated from regulated reactions, the molecules and the reactions are represented as typed nodes, as shown in Figure 1.

Thus, we propose to extend the BioPAX toolbox with a Python library able to interpret BioPAX files as graphs of regulated reactions. With PAX2GRAPHML, the graphs are represented in the .graphml format, allowing the manipulation of nodes and edges properties. The PAX2GRAPHML tool also enables extracting sub-graphs, by filtering the original files according to specific properties of the nodes (genes or proteins) or by merging different graphs. It also implements basic methods to explore the graphs. Thanks to the .graphml exchange format support, generated graphs can be further analyzed with already existing graph libraries in Python or R.

## 2 Format and package description

PAX2GRAPHML is able to process all BioPAX files to generate regulated reaction graphs, which can be further interpreted into positive and negative oriented influences. It is available on pypi and as a docker image.

In PAX2GRAPHML, PaxTools (Babur *et al.*, 2014) is used internally to extract sub-classes of patterns and further interpret them as regulated reactions. These extracted patterns form the building elements of a *regulated reaction graph* (Blavy *et al.*, 2014). Each regulated reaction graph pattern is centered on a reaction node linked to one or several substrate nodes and product nodes. The reaction node can also be linked to modulator nodes (activators or inhibitors). Substrates and modulators are inputs of the reaction node, whereas products are outputs of the reaction node. All nodes (reaction, substrate, product or modulator) are associated with their own metadata in the graph.

PAX2GRAPHML is composed of four sub-packages. (i) The sub-package pax_import is dedicated to global or parametrized import of BioPAX files from Pathway Commons (PC) to be further interpreted as regulated reaction graph. (ii) The sub-package properties allow to manipulate nodes and edges properties of the generated graphs. All aliases contained in BioPAX have been incorporated in the .graphml format as node properties to represent genes, protein and compounds. Additional annotations can also be directly imported from specific files. (iii) The sub-package extract allows modifying either the generated reaction graph or the influence graph, including sub-graphs selection or graphs merging. (iv) The sub-package graph_explore includes IO functions and analysis of the generated graphs. It also includes classical graph metrics (degree, betweenness, closeness, connected components) as preliminary steps. More sophisticated analyses can be further performed with graph-tool or other advanced libraries (Csardi and Nepusz, 2006).

The PAX2GRAPHML website provides a complete documentation and the pre-processed database resources. Regulated reaction graphs and influence graphs produced from 16 data sources of PC can be downloaded as ready-to-use data for further analyses with PAX2GRAPHML. Files are automatically updated using databanks synchronization and a processing software (Filangi *et al.*, 2008).

## 3 Application

PAX2GRAPHML was first applied to the complete PC databank. The regulated reaction graph produced in.graphml format has a size of 363 MB (13% of the initial BioPAX file size). PAX2GRAPHML was also applied to each data source of PC considered independently. As shown in Table 1, the regulated reaction concept used to unify the different BioPAX reaction types facilitates the comparison of the content of each resource. Notably, this revealed that Mirtarbase and CTD are the main contributors of PC in terms of nodes, edges, and especially inhibition reactions.

**Table 1.** BioPAX files transformation of datasources available in PC into regulated reaction graphs with PAX2GRAPHML

Data sources	Nodes	Reaction nodes	Entity nodes	Edges	Substrate of	Product of	Activator of	Inhibitor of
PC^*^ all sources	175 262	85 750	89 512	639 945	84 496	95 743	52 009	407 697
CTD	44 639	19 814	24 825	98 993	18 538	19 073	35 077	26 305
HumanCyc	5733	1778	3955	11 890	3875	4455	3560	0
INOH	4315	2188	2127	7409	4247	3162	0	0
Intact complex	2187	563	1624	2869	2306	563	0	0
KEGG^**^	3133	1560	1573	7041	3488	3553	0	0
Mirtarbase	32 727	15 064	17 663	395 703	0	15 064	0	380 639
Panther	3766	1662	2104	5263	2914	2165	142	42
PID	9403	4495	4908	14 827	6613	4544	3233	437
Reactome	31 718	11 404	20 314	46 541	27 210	15 358	3717	256
Reconx	6956	2821	4135	20 485	7722	7689	5074	0
PC^*^ all sources except CTD and Mirtarbase	119 285	55 184	64 101	167 515	83 782	66 056	16 939	7 38
PID and HumanCyc	12 561	4518	8043	16 085	3575	5859	6221	430
PID and HumanCyc and KEGG	15 564	6079	9485	19 651	5332	7652	6237	430
PID and HumanCyc and KEGG and Reactome	38 585	10 398	28 187	43 899	16 087	17 371	9756	685

Note: Single datasources transformations were performed with the sub-package pax_import. Combination of several datasources was performed by filtering the PC* all sources graphml file with the sub-package extract. Nodes are either reactions or entities (proteins, small molecules, etc.). The numbers of regulated reactions computed by PAX2GRAPHML, together with the number of substrates, products, activators, inhibitors, are indicated. All these graphs can be directly downloaded from the PAX2GRAPHML website.

* ‘PC' version 12, September 2019.

** KEGG, July 2011 (only human, hsa* files).

Generating the regulated reaction graph from 16 BioPAX datasources with PAX2GRAPHML lasted 7 days on a virtual machine with 48 G RAM. Conveniently, the generated files can be downloaded on PAX2GRAPHML website as ready-to-use data resources, which is automatically updated.

Customized graphs can be produced for any subsets of the databases. To achieve this, users can either filter the overall regulated reaction graph, or can merge the regulated reaction graphs produced from two or more databases selected according to their specific interest. The two functionalities (filtering and merging) are available within the PAX2GRAPHML package. As an illustration, Table 1 shows that filtering out CTD and Mirtarbase from PC eliminates 32% of the nodes (36% of reaction nodes and 28% of entity nodes) and 74% of the edges. Table 1 also illustrates that the combination of PID with successively HumanCyc, KEGG and Reactome improves coverage of both reaction nodes (from 4495 to 10 398) and entities (from 4908 to 28 187).

By managing BioPAX data extraction into regulated graphs, PAX2GRAPHML simplifies the implementation of many methods for regulation network analysis and understanding of the controlling steps of the biological pathways.

*Financial Support*: none declared.

*Conflict of Interest*: none declared.

## References

[btab441-B1] Babur O. et al (2010) ChiBE: interactive visualization and manipulation of BioPAX pathway models. Bioinformatics, 26, 429–431. 20007251 10.1093/bioinformatics/btp665 PMC2815657

[btab441-B2] Babur O. et al (2014) Pattern search in BioPAXmodels. Bioinformatics, 30, 139–140. 24045775 10.1093/bioinformatics/btt539 PMC3866551

[btab441-B3] Blavy P. et al (2014) Using a large-scale knowledge database on reactions and regulations to propose key upstream regulators of various sets of molecules participating in cell metabolism. BMC Syst. Biol., 8, 32. 24635915 10.1186/1752-0509-8-32 PMC4004165

[btab441-B4] Csardi G. , Nepusz T. (2006) The igraph software package for complex network research. InterJ. **Complex Syst.**, 1695, 1–9.

[btab441-B5] Filangi O. et al (2008) BioMAJ: a flexible framework for databanks synchronization and processing. Bioinformatics (Oxford, England), 24, 1823–1825. 18593718 10.1093/bioinformatics/btn325 PMC3293366

[btab441-B6] Rodchenkov I. et al (2013) The BioPAX validator. Bioinformatics, 29, 2659–2660. 23918249 10.1093/bioinformatics/btt452 PMC3789551

[btab441-B7] Shannon P. et al (2003) Cytoscape: a software environment for integrated models of biomolecular interaction networks. Genome Res., 13, 2498–2504. 14597658 10.1101/gr.1239303 PMC403769

[btab441-B8] Turei D. et al (2016) OmniPath: guidelines and gateway for literature-curated signaling pathway resources. Nat. Methods, 13, 966–967. 27898060 10.1038/nmeth.4077

